# Comparison of Diagnosis-Specific Survival Scores for Patients with Small-Cell Lung Cancer Irradiated for Brain Metastases

**DOI:** 10.3390/cancers11020233

**Published:** 2019-02-16

**Authors:** Dirk Rades, Heinke C. Hansen, Stefan Janssen, Steven E. Schild

**Affiliations:** 1 Department of Radiation Oncology, University of Lübeck, 23562 Lübeck, Germany; heinke_hansen@web.de (H.C.H.); st-janssen@gmx.net (S.J.); 2 Medical Practice for Radiotherapy and Radiation Oncology, 30161 Hannover, Germany; 3 Department of Radiation Oncology, Mayo Clinic, Scottsdale, AZ 85259, USA; sschild@mayo.edu

**Keywords:** brain metastasis, small-cell lung cancer, whole-brain radiotherapy, diagnosis-specific survival scores, positive predictive values

## Abstract

Diagnosis-specific survival scores including a new score developed in 157 patients with brain metastases from small-cell lung cancer (SCLC) receiving whole-brain radiotherapy (WBRT) with 30 Gy in 10 fractions (WBRT-30-SCLC) were compared. Three prognostic groups were designed based on the 6-month survival probabilities of significant or almost significant factors, (age, performance score, number of brain metastases, extra-cerebral metastasis). Six-month survival rates were 6% (6–11 points), 44% (12–14 points) and 86% (16–19 points). The WBRT-30-SCLC was compared to three disease-specific scores for brain metastasis from SCLC, the original and updated diagnosis-specific graded prognostic assessment DS-GPA classifications and the Rades-SCLC. Positive predictive values (PPVs) used to correctly predict death ≤6 months were 94% (WBRT-30-SCLC), 88% (original DS-GPA), 88% (updated DS-GPA) and 100% (Rades-SCLC). PPVs to predict survival ≥6 months were 86%, 75%, 76% and 100%. For WBRT-30-SCLC and Rades-SCLC, differences between poor and intermediate prognoses groups and between intermediate and favorable prognoses groups were significant. For both DS-GPA classifications, only the difference between poor and intermediate prognoses groups was significant. Of these disease-specific tools, Rades-SCLC appeared to be the most accurate in identifying patients dying ≤6 months and patients surviving ≥6 months after irradiation, followed by the new WBRT-30-SCLC and the DS-GPA classifications.

## 1. Introduction

Various radiotherapy techniques are available for the treatment of brain metastasis including whole-brain radiotherapy (WBRT), stereotactic radiosurgery (SRS) and fractionated stereotactic radiotherapy (FSRT) [1]. The use of SRS or FSRT alone is generally limited to patients with a maximum of 3–5 intracerebral lesions, particularly in case of less radiosensitive primary tumors such as malignant melanomas and renal cell carcinomas. During recent years these local therapies have even been used for up to 10 lesions [2]. However, despite these new developments, WBRT alone is still considered appropriate for many patients with brain metastases, particularly for patients with more than 3–5 lesions or in case of significant comorbidities or a poor performance status [1]. In comparison to other primary tumors, WBRT is frequently used for brain metastasis from small-cell lung cancer (SCLC), since this tumor tends to spread to the brain, resulting in multiple cerebral lesions. Even if brain metastases are not present, many patients with SCLC receive WBRT as prophylactic cranial irradiation [3]. When a patient with brain metastasis from SCLC receives WBRT, a variety of dose-fractionation schedules are available including short-course WBRT with 20 Gy in 5 fractions of 4 Gy and the longer-course schedules such as 30 Gy in 10 fractions and 40 Gy in 20 fractions [1]. 

When selecting the most appropriate schedule for a specific patient, the patient’s remaining lifespan should be taken into consideration. Patients with a short lifespan are good candidates for short-course WBRT, spending the least time possible receiving treatment to use the remaining lifetime for other important issues. Short-course WBRT is a reasonable option for these patients, since it is as effective as longer-course schedules in patients with multiple lesions and limited prognoses [4]. Since the risk of neuro-cognitive deficits caused by WBRT increases with lifetime, patients with intermediate or favorable survival prognoses appear to be better treated with lower doses per fraction that were reported to result in less neuro-cognitive decline than higher doses per fraction [5,6]. In addition, the risk of neuro-cognitive deficits can be reduced with the modern technique of hippocampal sparing WBRT and the administration of memantine [7,8,9]. Patients with very favorable prognoses may additionally benefit from total doses >30 Gy in terms of better intracerebral control and survival [10]. 

Scoring tools for estimating the survival of patients with brain metastasis were created to help physicians choosing the best individual WBRT schedule [11,12,13,14]. Such scoring tools included the recursive partitioning analysis (RPA) classification published in 1997 and the graded prognostic assessment (GPA) classification from 2008 [11,12]. Both classifications were designed from heterogeneously treated patient cohorts. Treatments included different WBRT programs, combinations of WBRT and misonidazole or chemotherapy, and in case of the GPA classification also WBRT plus a SRS boost. Thus, hidden selection biases may have been introduced when creating the scoring tools as these were created retrospectively. Therefore, we presented another score in 2008 that was obtained from patients treated with WBRT alone [13]. Additionally, different WBRT schedules were included and the risk of a hidden bias (although to a lesser extent) remained. 

To significantly reduce the risk of a bias due to the different treatments used, we created the WBRT-30 published in 2013 [14]. In contrast to the previous scores, the cohort of patients used for the WBRT-30 was uniformly treated with WBRT alone using 30 Gy in 10 fractions. The positive predictive values (PPVs) of the WBRT-30 to identify patients dying ≤6 months and those surviving ≥6 months were quite high (97% and 96%), demonstrating a high level of accuracy [14]. These PPVs were higher than for the RPA classification (92% and 75%), the GPA classification (85% and 64%) and our previous score (96% and 73%) [11,12,13]. Therefore, the WBRT-30 could be recommended when aiming to predict death within 6 months or survival for at least 6 months following WBRT. 

In order to account for the different biology of tumor entities associated with brain metastases and to further improve the personalization of the treatment for these patients, separate scoring tools for each tumor type are important. Such tools are already available for patients with brain metastasis from SCLC including the original diagnosis-specific graded prognostic assessment (DS-GPA) classifications, the updated diagnosis-specific graded prognostic assessment DS-GPA classification and the Rades-SCLC score. In addition, significant prognostic factors were identified in a cohort of 229 patients with brain metastases from SCLC including performance status, time of appearance of brain metastases, initial response to chemotherapy and RPA-class [15]. In this study, 95% of the patients received WBRT with 30 Gy in 10 fractions, which likely led to a significant reduction of hidden selection biases. However, no scoring tool was created from the significant prognostic factors [15]. In the present study, another diagnosis-specific score for patients with brain metastases from SCLC was developed. Considering the high PPVs to correctly identify patients dying within 6 months and patients living for at least 6 months of the (general) WBRT-30 [14] the new diagnosis-specific WBRT-30-SCLC was also created from patients uniformly treated with 30 Gy in 10 fractions of WBRT alone. 

Moreover, the new WBRT-30-SCLC was compared to the three existing tools created for patients irradiated for brain metastases from SCLC with respect to correct identification of patients dying ≤6 months and patients surviving ≥6 months after irradiation.

## 2. Results

### 2.1. Development of the WBRT-30-SCLC

In the entire cohort, the survival rates at 3, 6, 9 and 12 months were 50%, 29%, 23% and 17%, respectively. On univariate analysis (Table 1), significant associations with better survival were found for age ≤64 years (*p* = 0.002), Karnofsky performance score (KPS) >70 (*p* < 0.001), 1–3 cerebral lesions (*p* < 0.001) and no extra-cerebral metastasis at WBRT (*p* = 0.004). A trend towards better survival was found for systemic treatment prior to WBRT (*p* = 0.07) and controlled primary tumor (*p* = 0.09). Gender and interval from diagnosis of SCLC to WBRT (*p* = 0.71) were not significantly associated with survival.

In the Cox regression analysis, age (risk ratio [RR]: 1.66; 95%-confidence interval [CI]: 1.17–2.35; *p* = 0.004), KPS (RR: 1.91; 95%-CI: 1.52–2.41; *p* < 0.001) and number of cerebral lesions (RR: 1.21; 95%-CI: 1.06–1.39; *p* = 0.005) were significant, and extra-cerebral metastasis (RR: 1.49; 95%-CI: 0.99–2.32; *p* = 0.058) was almost significant.

**Figure 1:**
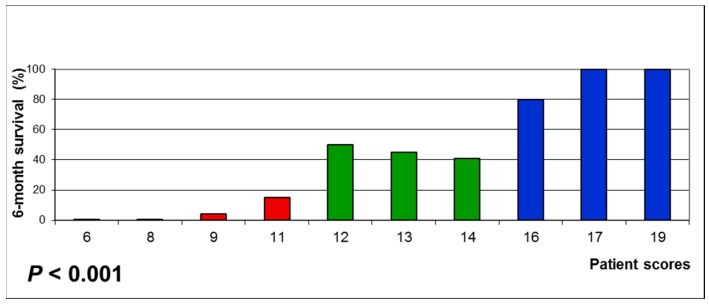
Six-month survival rates of the patient scores used for designing the prognostic groups of the new score developed in patients with brain metastases from small-cell lung cancer receiving whole-brain radiotherapy with 30 Gy in 10 fractions (WBRT-30-SCLC). A *p*-value of <0.05 was considered significant.

**Figure 2:**
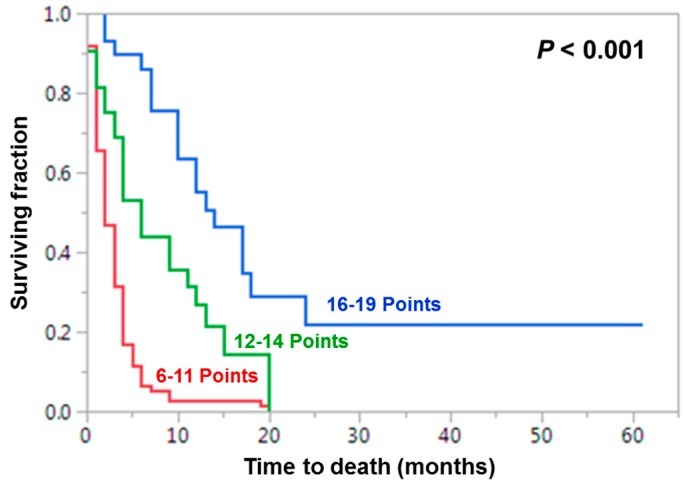
Kaplan-Meier curves of the three prognostic groups of the WBRT-30-SCLC. A *p*-value of <0.05 was considered significant.

These factors were used to design the WBRT-30-SCLC as described in the Materials and Methods section. Factor scores are shown in Table 2. After adding the factors scores for each patient, patient scores were received that ranged between 6 and 19 points (Figure 1). The 6-month survival rates of the patient scores led to three prognostic groups: 6-11 points (*n* = 96), 12-14 points (*n* = 32) and 16–19 points (*n* = 29). The 6-month survival rates of these groups were 6%, 44% and 86% (*p* < 0.001, Figure 2).

### 2.2. Comparison of Four Disease-Specific Survival Scores for Patients with Brain Metastasis from SCLC

The prognostic groups of the WBRT-30-SCLC and the other three scores are shown in Table 3. The positive predictive value (PPV) of the least favorable prognostic group (6–11 points) of the new WBRT-30-SCLC to correctly identify patients dying ≤6 months after irradiation was 94%. The corresponding PPVs were 88% in patients with the least favorable score of 0.0–1.0 in both the original and the updated DS-GPA classifications for SCLC and 100% in the least favorable group (5–8 points) of our previous score (Rades-SCLC) [16,17,18]. The PPV of the most favorable group (16–19 points) of the WBRT-30-SCLC to identify patients surviving ≥6 months after irradiation was 86%. The corresponding PPVs were 75% for a score of 3.0–4.0 in the original DS-GPA, 76% for a score of 2.5–4.0 in the updated DS-GPA classification, and 100% in the Rades-SCLC. DS-GPA scores of 3.0–4.0 and 2.5–4.0 were chosen, since only two patients had a score >3.0 [16,17].

For the WBRT-30-SCLC, the differences between the 6-11 points (poor prognoses) and the 12–14 points (intermediate prognoses) groups (*p* < 0.001) and between the 12–14 points and the 16–19 points (favorable prognoses) groups (*p* = 0.037) were significant. Also for our previous Rades-SCLC score, the differences between the 5–8 points (poor prognosis) and the 9–12 points (intermediate prognosis) groups (*p* < 0.001) and between the 9–12 points and the 15 points (favorable prognosis) groups (*p* = 0.008) were significant. For the both the original and the updated DS-GPA scores, the difference between 0.0–1.0 and 1.5–2.0 was significant (*p* < 0.001 and *p* < 0.001, respectively), but the differences between 1.5–2.0 and 3.0–4.0 in the original DS-GPA classification (*p* = 0.59) and between 1.5–2.0 and 2.5–4.0 in the updated DS-GPA classification (*p* = 0.33) were not [16,17]. 

Thus, of the four compared disease-specific tools, the Rades-SCLC appeared to be the most accurate in identifying both patients dying ≤6 months and patients surviving ≥6 months after irradiation, followed by the new WBRT-30-SCLC and the two DS-GPA classifications [16,17,18].

## 3. Discussion

If a patient has developed brain metastases, the situation is palliative and personalized treatment regimens are required to optimally meet an individual patient’s needs [1,19,20]. If the decision has been made that the patient should receive WBRT, it is important to select the appropriate doe-fractionation regimen to avoid under-or overtreatment. Patients with very favorable survival prognoses can benefit from longer-course WBRT with total doses >30 Gy. A previous study compared 40 Gy in 20 fractions to 30 Gy in 10 fractions in 186 patients with favorable survival prognoses [10]. The 1-year intracerebral control rates were 44% and 28% (*p* = 0.047 on multivariate analysis), and the 1-year survival rates 61% and 50% (*p* = 0.008 on multivariate analysis). Moreover, doses per fraction >3.0–3.5 Gy were suggested to result in greater neuro-cognitive deficits than lower doses per fraction [5,6]. Since the risk of developing a decline in neuro-cognitive function is positively correlated with lifetime, patients with more favorable survival prognoses should receive lower doses per fraction. The risk of a WBRT-induced decline in neuro-cognitive function can be further reduced with hippocampal sparing [7,8]. In a previous phase 2 trial, decline in neuro-cognitive function at 4 months following WBRT (30 Gy in 10 fractions) was 7% in patients receiving hippocampal sparing versus 30% in a historical control group (*p* < 0.001) [7]. In an additional study of patients with SCLC receiving prophylactic or therapeutic WBRT, 59 patients experienced new brain metastases and 20 patients a progression of treated lesions [8]. Of this series, only 3 patients (5%) and 1 patient (5%), respectively, had new or progressive lesions in the hippocampal avoidance region. Therefore, hippocampal sparing appears to be a reasonable option for patients with brain metastasis from SCLC. Another option to reduce neuro-cognitive deficits is the administration of memantine [9]. In a randomized, placebo-controlled trial of 554 patients receiving WBRT plus/minus memantine, the interval to neuro-cognitive decline was significantly longer in the memantine group (*p* = 0.01), and the probability of neuro-cognitive dysfunction at 24 weeks was 54% with and 65% without memantine [9]. On the other hand, for patients with a poor expected survival, WBRT with 20 Gy in 5 fractions is considered more appropriate, since it is similarly effective for intracerebral control and survival as longer-course WBRT schedules and is not associated with increased acute toxicity [1,4]. Selected patients may also be considered for best supportive care (BSC) including corticosteroids. In a randomized trial of patients with brain metastases from non-small cell lung cancer and a very poor survival prognosis, treatment with BSC and dexamethasone was not inferior to BSC and dexamethasone plus short-course WBRT with 20 Gy in 5 fractions [21,22]. 

It is very important to be able to judge a patient’s survival prognosis before assigning a WBRT schedule. To support the treating physicians, several prognostic tools were created [11,12,13,14]. 

In order to provide optimal personalization of the treatment including the most appropriate WBRT schedule, diagnosis-specific tools would be desirable taking into account the differences of the various primary tumor types with respect to biological behavior and prognoses. Diagnosis-specific classifications for brain metastasis from SCLC already exist, including an original and an updated DS-GPA and our previous score including two different WBRT regimens [16,17,18]. Since the PPVs of the WBRT-30 were quite promising, we decided to create a diagnosis-specific WBRT-30 particularly for patients with SCLC, the WBRT-30-SCLC. 

This new tool included three prognostic groups with significantly different 6-month survival probabilities. When compared to the original and the updated DS-GPS for SCLC, the PPV of the WBRT-30-SCLC to correctly identify patients dying ≤6 months following irradiation was slightly higher (94% vs. 88%). However, it was slightly lower than with the previous Rades-SCLC score (94% vs. 100%). When aiming to identify patients surviving ≥6 months, the PPV of the WBRT-30-SCLC was 11% and 10% higher than with the DS-GPA classifications (86% vs. 75% and 76%) but was 14% lower than with the Rades-SCLC (86% vs. 100%). 

Moreover, the differences in 6-month survival between the poor-prognosis and the intermediate-prognosis group and between the intermediate-prognosis and the favorable-prognosis group of the WBRT-30-SCLC were significant. So were the differences when using the Rades-SCLC [18]. In contrast, when applying both DS-GPA classifications, only the difference between the poor-prognosis and the intermediate-prognosis group was significant [16,17]. Thus, the WBRT-30-SCLC appeared more accurate than the original and the updated DS-GPA classifications in identifying patients surviving ≥6 months. Such superiority was already shown for the general WBRT-30 when compared to the general GPA classification (PPV 96% vs. 64%) [14]. The general WBRT-30 was also more accurate in identifying patients surviving ≥6 months than the general Rades-score developed in patients receiving different WBRT-schedules (PPV 95% vs. 73%) [13]. Surprisingly the WBRT-30-SCLC appeared to be less accurate than the previous Rades-SCLC, although patients included in the WBRT-30-SCLC received a more homogeneous treatment and the data, therefore, had a lower risk of being influenced by a selection bias. This finding supports the idea of different behavior of various solid tumors such as SCLC. 

The WBRT-30-SCLC needs to be validated in a separate cohort of patients in the future because advances in systemic therapies, especially the development immuno-therapies that can cross the blood-brain-barrier will have an impact on the survival of patients with brain metastases from lung cancer. Promising results were reported for immune checkpoint inhibitors or alk inhibitors in patients with brain metastases from non-small cell lung cancer [23,24,25,26]. Moreover, a phase 2 study of maintenance pembrolizumab in 45 patients with extensive disease SCLC, of whom 22% had treated brain metastases, suggested that a subset of these patients could benefit from this treatment [27].

## 4. Materials and Methods

In order to create a new disease-specific survival score (WBRT-30-SCLC) for patients with brain metastasis from SCLC, eight factors were evaluated for potential impact on survival in a retrospective cohort of 157 patients receiving WBRT alone with 30 Gy in 10 fractions between 1998 and 2017 (Table 4). The study was approved by the ethics committee of the University of Lübeck (reference: 19-003A). Univariate analyses of survival were performed using the Kaplan-Meier method and the log-rank test. Significant factors (*p* < 0.05) were included in a Cox regression analysis. Factors achieving significance (*p* < 0.05) or almost significance (*p* < 0.06) in the Cox regression analysis were used for the WBRT-30-SCLC. Factor scores were obtained from the 6-month survival rates divided by 10 and patient scores by addition of the factor scores for each patient. The patient scores were used for designing the prognostic groups.

The WBRT-30-SCLC and three other disease-specific scores for SCLC were compared with respect to positive predictive values (PPV) for correctly predicting death ≤6 months and survival ≥6 months after irradiation [16,17,18]. The other scores included the original and the updated diagnosis-specific graded prognostic assessment (DS-GPA) classifications for SCLC (Table 5) and our previous score (Rades-SCLC) created in a cohort of patients receiving WBRT with 20 Gy in 5 fractions or 30 Gy in 10 fractions (Table 6). In addition, the discrimination between the prognostic groups was analyzed for each of the four scoring systems using the Chi-square test. 

## 5. Conclusions

A new diagnosis-specific tool (WBRT-30-SCLC) was created to estimate the remaining lifespan of patients irradiated for brain metastasis from SCLC. The WBRT-30-SCLC appeared to be superior to the original and the updated GPA classifications, particularly for identifying patients surviving ≥6 months. The WBRT-30-SCLC and both DS-GPA classifications appeared less accurate than the Rades-SCLC. Thus, the Rades-SCLC is the preferable tool when physicians aim to predict the survival prognosis of a patient with brain metastasis from SCLC that is planned to be treated with WBRT.

## Figures and Tables

**Table 1:** Results of the univariate analyses of survival of the factors that may be used to design the the new score developed in patients with brain metastases from small-cell lung cancer receiving whole-brain radiotherapy with 30 Gy in 10 fractions (WBRT-30-SCLC) including those factors that were significant (*p* < 0.05) or showed a trend (*p* < 0.10).

Factor	At 3 Months (%)	At 6 Months (%)	At 9 Months (%)	At 12 Months (%)	*p*-Value
Age					
≤ 64 years	60	38	33	22	
> 64 years	38	18	12	12	0.002
Karnofsky Performance Score					
< 70	20	0	0	0	
= 70	52	34	28	28	
> 70	76	52	42	28	<0.001
Systemic treatment prior to WBRT					
No	38	22	20	17	
Yes	55	32	24	17	0.07
Controlled primary tumor					
No	43	28	21	14	
Yes	55	29	25	21	0.09
Number of brain metastases					
1–3	67	54	50	40	
≥ 4	42	18	11	7	<0.001
Extra-cerebral metastasis					
No	58	50	41	39	
Yes	47	22	17	10	0.004

**Table 2:** Factor scores used for creating the WBRT-30-SCLC.

Factor	6-Month Survival Rate (%)	Factor Score
Age		
≤ 64 years	38	4
> 64 years	18	2
Karnofsky Performance Score		
< 70	0	0
= 70	34	3
> 70	52	5
Number of brain metastases		
1–3	54	5
≥ 4	18	2
Extra-cerebral metastasis		
No	50	5
Yes	22	2

**Table 3:** Prognostic groups of WBRT-30-SCLC, Rades-SCLC and original and updated diagnosis-specific graded prognostic assessment (DS-GPS) classifications [16,17,18].

ColuPrognostic Group	WBRT-30-SCLC		Rades-SCLC		Original DS-GPA		Updated DS-GPA	
	Scoring points	6-month Survival	Scoring points	6-month survival	Scoring points	6-month survival	Scoring points	6-month survival
Poor prognosis	6–11	6% (6/96)	5–8	0% (0/59)	0.0–1.0	12% (12/103)	0.0–1.0	12% (12/103)
Intermediate prognosis	12–14	44% (14/32)	9–12	40% (35/88)	1.5–2.5	59% (27/46)	1.5–2.0	54% (20/37)
Favorable prognosis	16–19	86% (25/29)	15	100% (10/10)	≥ 3.0	75% (6/8)	≥ 2.5	76% (13/17)

**Table 4:** Distribution of the potential prognostic factors evaluated for potential inclusion in the WBRT-30-SCLC.

Factor	N Patients (%)
Age	
≤ 64 years	81 (52)
> 64 years	76 (48)
Gender	
Female	60 (38)
Male	97 (62)
Karnofsky Performance Score	
< 70	61 (39)
= 70	29 (18)
> 70	67 (43)
Interval from diagnosis of	
SCLC to WBRT	
≤ 2 months	80 (51)
> 2 months	77 (49)
Systemic treatment prior to WBRT	
No	50 (32)
Yes	107 (68)
Controlled primary tumor	
No	74 (47)
Yes	83 (53)
Number of brain metastases	
1–3	46 (29)
≥ 4	111 (71)
Extra-cerebral metastasis	
No	38 (24)
Yes	119 (76)

**Table 5:** Diagnosis-specific graded prognostic assessment (DS-GPA) classification for patients with brain metastasis from SCLC [16,17].

Colu Factor	GPA Scoring Criteria		
	0	0.5	1.0
Age (years)	>60	50–60	<50
Karnofsky Performance Score	<70	70-80	90–100
Extra-cerebral metastasis	present	----	absent
Number of brain metastases	>3	2–3	1

Prognostic groups of the original DS-GPA: 0.0–1.0, 1.5–2.5, 3.0, 3.5–4.0, higher scores = better prognoses [16]. Prognostic groups of the updated DS-GPA: 0.0–1.0, 1.5–2.0, 2.5–3.0, 3.5–4.0, higher scores = better prognoses [17].

**Table 6:** Scoring points of the previous Rades-SCLC score [18].

Factor	6-Month Survival Rate (%)	Factor Score
Karnofsky Performance Score		
< 70	5	1
≥ 70	51	5
Number of brain metastases		
1–3	54	5
≥ 4	18	2
Extra-cerebral metastasis		
No	50	5
Yes	22	2

Prognostic groups of the Rades-SCLC: 5–8 points, 9–12 points, 15 points, higher scores = better prognoses [17].
